# In Vitro Assessment of 3T MRI Effects on Non–3T‐Compatible Cardiac Implantable Electronic Devices

**DOI:** 10.1111/jce.70397

**Published:** 2026-06-07

**Authors:** Yu Fukuoka, Yasuo Sakurai, Risa Kanai, Yuuya Miyazaki, Haruka Yamazaki, Yusuke Koga, Isao Tsukamoto, Yoshifumi Ikeda, Hitoshi Mori, Kazuhisa Matsumoto, Ritsushi Kato

**Affiliations:** ^1^ Clinical Engineering Department Saitama Medical University International Medical Center Hidaka, Saitama Japan; ^2^ Central Radiology Department Saitama Medical University, International Medical Center Hidaka, Saitama Japan; ^3^ Outpatient Clinic of Cardiology Saitama Medical University, International Medical Center Hidaka, Saitama Japan; ^4^ Department of Cardiology Saitama Medical University, International Medical Center Hidaka, Saitama Japan

**Keywords:** 3 Tesla MRI, cardiac implantable electronic device, magnet mode, pacemaker, telemetry failure

## Abstract

**Background:**

The safety of cardiac implantable electronic devices (CIEDs) during 3‐Tesla (3T) MRI remains uncertain, particularly for devices not designed for 3T use. We experimentally evaluated 3T MRI effects on 3T‐compatible and non–3T‐compatible CIEDs.

**Methods:**

Fifty‐eight CIEDs (37 pacemakers, 21 ICDs; 3T MRI‐conditional *n* = 31, 1.5‐T MRI‐conditional *n* = 14, and non–MRI‐conditional *n* = 13) were tested in a 3T scanner. Each device was immersed in saline with connected leads and programmed to asynchronous or demand pacing with MRI mode ON or OFF. Device interrogation before and after scanning assessed telemetry integrity, parameter retention, and pacing‐mode changes.

**Results:**

Most CIEDs completed MRI without malfunction and remained telemetric. However, among pacemakers, telemetry failure or parameter reset occurred in 6 non–MRI‐conditional and 3 1.5‐T MRI‐conditional devices, including some devices with MRI mode ON. Reset or telemetry failure was more frequent in devices with low battery status (≤ 30% to ERI) than in those with higher status (6/11 [55%] vs. 3/22 [14%], *p*= 0.013). With MRI mode OFF, magnet mode was activated in 11 3T and 7 non–MRI‐conditional pacemakers; activation frequency did not differ between asynchronous and demand pacing (16/31 [52%] vs. 18/31 [58%], *p*= 0.610). In some pacemakers and ICDs, demand pacing led to atrial mode switch or pacing inhibition, but no reset or telemetry failure occurred in ICDs.

**Conclusions:**

In direct in vitro 3T MRI exposure experiments involving non–3T MRI CIEDs, some pacemakers approaching ERI demonstrated an increased risk of telemetry failure or reset, and those with MRI mode turned off showed non‐negligible mode switching. However, the majority of devices did not exhibit severe electrical malfunctions necessitating replacement in a clinical setting, and no instances of device reset or telemetry failure were observed in ICDs.

**Abbreviations**
AMS: atrial mode switch
AVD: atrioventricular delay time
CIEDs: cardiac implantable electronic devices
ICD: implantable cardioverter defibrillator
MRI: magnetic resonance imaging
PM: pacemaker

## 1 Introduction

With the increasing global adoption of 3‐Tesla (3T) magnetic resonance imaging (MRI) in clinical practice, most newly implanted cardiac implantable electronic devices (CIEDs) are now 3T MRI‐ compatible. However, a substantial number of patients still have older CIEDs systems that are not compatible with 3T MRI scanners.

Many large‐scale studies [1, 2, 3, 4] have demonstrated the feasibility of performing MRI in patients with non–MRI‐conditional CIEDs, as well as in cohorts that include patients with MRI‐conditional CIEDs. In the current HRS statement [5] and ESC guidelines [6], MRI scanning is classified as a Class IIa indication when the leads are connected to the device, even if the device itself is non–MRI‐conditional. However, even in reports describing MRI scans as being performed safely, a small number of reset and telemetry failure events have in fact also been reported [1, 2, 3]. Further, most reported MRI experiences have been limited to 1.5 T MRI [7, 8]. A Meta‐analysis has shown that when MRI is performed at field strengths exceeding 1.5 T, the composite clinical safety event rate is significantly higher, with a hazard ratio of approximately 5.96 compared with 1.5 T [7]. Most of these adverse events were attributed to power‐on reset, occasionally leading to pacing inhibition or even cardiac arrest, including in a case with a “modern” CIED system [9]. These findings indicate that the safety of 3T MRI—particularly in patients with non‐MRI‐conditional CIEDs—remains uncertain and warrants further investigation. Moreover, although previous reports [10, 11] in 3T MRI describe the post‐scan status of the device, few have addressed the behavior of atrial mode switch (AMS) or magnet mode during the MRI examination itself. In addition, the initial multicenter large‐scale trial, the MagnaSafe study [1], excluded chest imaging in which the device is located close to the scanner's centerline. Therefore, this study was conducted to directly scan non–MRI‐conditional, 1.5T MRI‐conditional, and 3T MRI‐conditional CIEDs and to clarify their device behaviors. In particular, although these were “modern devices,” the aim was to elucidate the electrical safety of 3T MRI scanning in non‐3T‐compatible devices.

## 2 Materials and Methods

### 2.1 The Types of CIEDs and Leads Connected to the Devices That Underwent MRI Scanning

Fifty‐eight CIEDs that had been explanted from patients for reasons such as infection or device upgrade were included in this study: 31 devices were 3T MRI conditional, 14 were 1.5T MRI conditional, and 13 were non‐MRI conditional (Tables 1, 2, 3). The leads used comprised two IS‐1 leads and one each of DF‐1, DF‐4, and SQ‐1 leads (Table 1). Additionally, devices with a programmer‐reported remaining battery level of ≤ 30% were classified as having a low battery status. Battery status was verified for each device using the manufacturer‐specific programmer. When a direct battery‐level value was not available, an approximate value was estimated from the reported remaining battery longevity. The methods used to estimate remaining battery longevity to the elective replacement indicator (ERI) differed by manufacturer, as follows: for Medtronic devices, an approximate value was calculated based on battery voltage and magnet rate; for Abbott (St. Jude Medical) and Biotronik devices, the programmer‐displayed percentage or ERI indicator was used; and for Boston Scientific and MicroPort CRM (Sorin) devices, the estimate was based on the displayed indicator, battery voltage, and magnet rate.

**Table 1:** Number of devices by MRI compatibility category.

		3.0T (*N *= 31)	1.5T (*N *= 14)	Non‐MRI (*N *= 13)	All (*N *= 58)
Device					
Medtronic (%)		12 (38.7)	0 (0)	1 (7.7)	13 (22.4)
Abbott (%)		7 (22.6)	8 (57.1)	4 (30.8)	19 (32.8)
Biotronik (%)		7 (22.6)	2 (14.3)	1 (7.7)	10 (17.2)
Boston Scientific (%)		3 (9.7)	4 (28.6)	1 (7.7)	8 (13.8)
MicroPort CRM (%)		2 (6.5)	0 (0)	6 (46.2)	8 (13.8)
Pacemaker					
Single Chamber (%)		2 (6.5)	0 (0)	2 (15.9)	4 (6.9)
Dual Chamber (%)		17 (54.8)	6 (42.9)	6 (46.2)	29 (50.0)
Leadless (%)		4 (12.9)	N/A	N/A	4 (6.9)
ICD					
Single Chamber (%)		0 (0)	1 (0)	0 (0)	1 (1.7)
Dual Chamber (%)		8 (25.8)	3 (21.5)	5 (38.5)	16 (27.6)
S‐ICD (%)		N/A	4 (28.6)	N/A	4 (6.9)
Battery status					
ERI > 30% (%)		28 (90.3)	4 (28.6)	6 (46.2)	38 (65.5)
ERI ≤ 30% (%)		3 (9.7)	10 (71.4)	7 (53.8)	20 (34.5)
Pacing lead type					
IS‐1	5076 58cm (Medtronic), 2088TC 52cm (Abbott)				
DF‐1, DF‐4	6947 (Medtronic), 6947M (Medtronic) SQ‐1 3501 45cm (Boston Scientific)				

**Table 2:** List of pacemakers.

Device *N *= 37	*N* (%)	Manufacturing year	Device *N *= 37	*N* (%)	Manufacturing year
**3T MRI conditional**			1.5T MRI conditional		
Medtronic			Abbott (St. Jude Medical)		
Azure XT DR, W2DR01	3 (8.1)	2023, 2025 (2)	Zenex MRI, PM2282	1 (2.7)	2019
Attesta S DR MRI, ATDRS1	1 (2.7)	2023	Accent MRI, PM2224	5 (13.5)	2013 (2), 2014 (3)
Advisa MRI, A3DR01	1 (2.7)	2015	non‐MRI conditional		
Micra VR, MC1VR01	1 (2.7)	2019	Medtronic		
Micra AV2, MC2AVR1	3 (8.1)	2024, 2025 (2)	Adapta DR, ADDR06	1 (2.7)	2018
Abbott (St. Jude Medical)			Abbott (St. Jude Medical)		
Assurity MRI, PM2272	6 (16.2)	2018, 2022 (2), 2023, 2024 (2)	ZEPHYR, 5826	1 (2.7)	2020
Boston Scientific			EMPRISE	1 (2.7)	2010
ACCOLADE, MRI L331	2 (5.4)	2025 (2)	MicroPort CRM (Sorin)		
Biotronik			Reply200 DR	4 (10.8)	2012 (2), 2013, 2018
Evity 8‐T pro MRI	3 (8.1)	2019 (3)	Reply200 SR	1 (2.7)	2015
Edora 8‐T pro MRI	1 (2.7)	2023			
MicroPort CRM (Sorin)					
Alizea DR1600	2 (5.4)	2023 (2)			

**Table 3:** List of ICDs.

Device *N* = 21	*N* (%)	Manufacturing year	Device *N *= 21	*N* (%)	Manufacturing year
**3T MRI conditional**			1.5T MRI conditional		
Medtronic			Boston Scientific		
Cobalt XT DR, DDPA2D4	2 (9.5)	2020, 2023	EMBLEM S‐ICD A209	2 (9.5)	2016(2)
Abbott (St. Jude Medical)			EMBLEM S‐ICD A219	2 (9.5)	2022, 2025
Gallant, CDHFA500Q	1 (4.8)	2023	non‐MRI conditional		
Boston Scientific			Abbott (St. Jude Medical)		
RESONATE, D433	1 (4.8)	2019	UNIFY, CD3235‐40	1 (4.8)	2012
Biotronik			UNIFY, CD3361‐40QC	1 (4.8)	2013
Acticor 7 DR‐T	2 (9.5)	2020, 2021	Boston Scientific		
Rivacor 7 HF‐T QP	1 (4.8)	2024	INCEPTA, F163	1 (4.8)	2015
Ilivia Neo VR‐T Pro MRI	1 (4.8)	2018	Biotronik		
**1.5T MRI conditional**			Inlexa7 HF‐T QP	1 (4.8)	2018
Abbott (St. Jude Medical)			MicroPort CRM (Sorin)		
QUADRA, CD3367‐40Q	2 (9.5)	2016, 2017	PLATINIUM, DR1540	1 (4.8)	2021
Biotronik					
Iperia 7 VR‐T	1 (4.8)	2015			
Iperia 7 DR‐T	1 (4.8)	2016			

### 2.2 CIED Configuration and Verification Parameters

**Figure 1:**
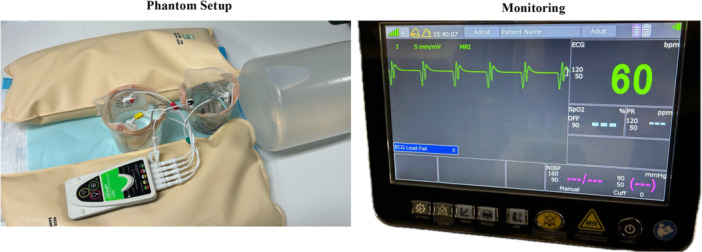
Phantom and monitoring setup used during MRI imaging. The container was filled with saline solution, and the cardiac implantable electronic devices (CIEDs) with connected leads were fully submerged. To satisfy the manufacturer‐defined lead‐impedance criterion required for activation of MRI mode, immersion was performed in 0.9% saline (200 mL per container) at approximately 10°C–20°C (room‐temperature range) prior to MRI‐mode programming/verification. This procedure was conducted solely to enable MRI‐mode programming and was not intended to reproduce physiological conditions (e.g., 37°C). Using an MRI‐compatible patient monitor (Pimot, IRADIMED, Orlando, FL), CIED behavior was observed before entering the MRI room, during imaging, and after exiting.

Each CIED was immersed in a saline‐filled container together with its corresponding lead, and device interrogation was performed using a programmer. To satisfy the manufacturer‐defined lead‐impedance criterion required for MRI‐mode activation, devices were immersed in 0.9% saline (200 mL per container) at approximately 10°C–20°C (room‐temperature range) before MRI‐mode programming and verification. This step was performed solely to enable MRI‐mode programming and was not intended to reproduce physiological conditions (e.g., 37°C). Under MRI mode OFF conditions, the devices were configured to asynchronous modes (DOO, VOO) and demand modes (DDD, VVI); they were also evaluated under MRI mode ON. Device behavior inside the MRI room was monitored using an MRI‐compatible patient monitoring system (Pimot, IRADIMED, Orlando, FL). In this system, the electrocardiogram is essentially a pseudo‐ECG that detects only pacing spikes; however, we could primarily assess the pacing rate in real time, including evidence of pacing inhibition or mode change. After each scan, the devices were re‐interrogated to confirm telemetry communication, review recorded episodes such as the ventricular pacing ratio, magnet response and the occurrence of atrial mode switching, and evaluate changes in battery status. In particular, Abbott pacemakers are equipped with a “magnet episode” function. Therefore, we defined events accompanied by a change in pacing rate on the electrocardiogram as “magnet rate pacing,” whereas those without a change in heart rate were defined as “magnet episodes”, when the device in the saline container was brought into the MRI room. Reprogramming and scanning were repeated for each configuration. For CIEDs equipped with defibrillation capability, the defibrillation function was kept OFF during verification (Figure 1).

**Figure 2:**
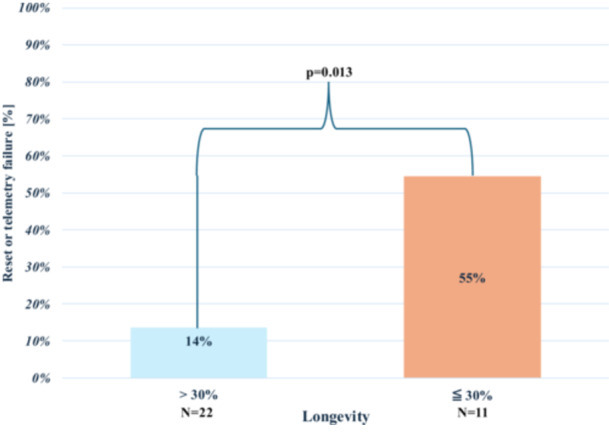
Transvenous pacemakers (*n* = 33) were stratified by the elective replacement indicator (ERI) margin using a battery longevity cutoff of 30% to the ERI. Among 22 devices with an ERI margin > 30%, 3 (14%) experienced parameter reset or telemetry failure, whereas among 11 devices with an ERI margin ≤ 30%, 6 (55%) did so, showing a significant difference between the two groups (*p* = 0.013).

**Figure 3:**
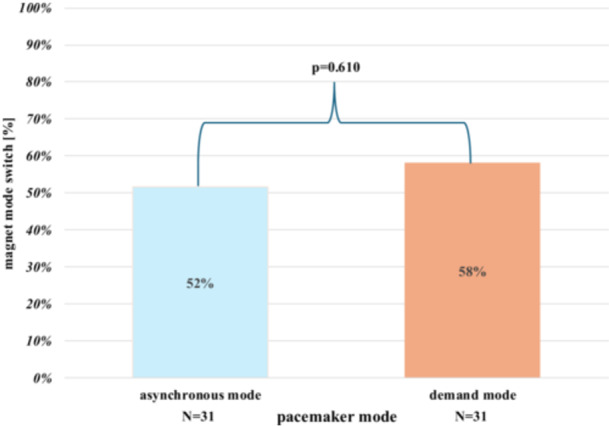
Switching to magnet mode was compared between asynchronous pacing (DOO/VOO) and demand pacing (DDD/VVI) with MRI mode OFF in pacemakers that completed all experimental protocols (*n* = 31). No significant difference in the frequency of magnet‐mode switching was observed between the two groups, although the operating characteristics and magnetic field intensity thresholds that triggered these changes differed among manufacturers (DOO/VOO vs. DDD/VVI: 16/31 [52%] vs. 18/31 [58%], *p* = 0.610).

In addition, pacing output (amplitude and pulse width) was kept at the nominal settings used in MRI mode ON for all pacing configurations. The lower rate limit was set to the device's nominal MRI mode ON value when the MRI mode was ON and to 60 ppm when the MRI mode was OFF. Sensitivity was programmed to the most sensitive setting (i.e., the lowest sensing threshold) available for each device.

### 2.3 3T MRI Imaging Protocol

All scans were performed using an Achieva dStream 3.0T (Philips) MRI system at our institution. The imaging protocol was selected with reference to routine head and thoracic MRI protocols used at our institute, and the scans were performed to directly image the pulse generator in order to maximize susceptibility to electromagnetic field effects. The protocol included T1 sequence (T1; SAR 0.8 W/kg; Max B1+rms 2.0 µT), T2 sequence (T2; SAR 0.6 W/kg; Max B1+rms 1.23 µT), and Balanced‐FFE (Balanced‐FFE; SAR 2.0 W/kg; Max B1+rms 1.2 µT). SAR and B1+rms values were recorded as displayed on the scanner console for each sequence.

To contextualize static magnetic field exposure during device transport and positioning within the MRI suite, the approximate field strength at the device location was estimated using the MRI system manufacturer's field distribution plot (fringe‐field/isogauss map). The field strength associated with magnet‐response events was recorded based on the device position relative to the isogauss contours.

### 2.4 Statistical Analysis

**Figure 4:**
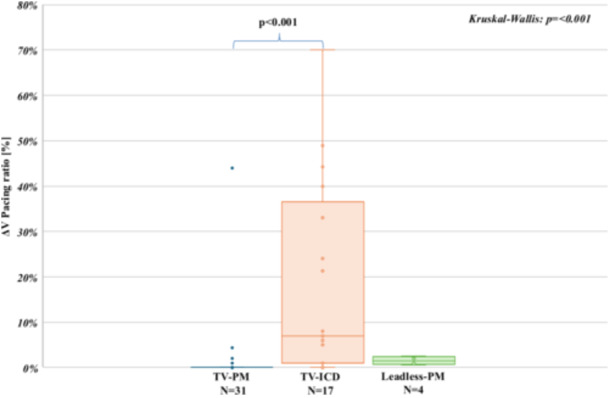
Data are presented as the median (center line), interquartile range (box), and 1.5 × IQR whiskers. Individual data points are overlaid with jitter. Overall differences among groups were assessed using the Kruskal–Wallis test, followed by Dunn's post hoc test with a Holm adjustment for multiple comparisons. Ventricular pacing suppression was minimal in transvenous pacemakers (TV‐PM) and leadless pacemakers, whereas it was greater in transvenous ICDs (TV‐ICD). Accordingly, the degree of suppression differed significantly among the three groups, with a significant difference between TV‐PMs and TV‐ICDs.

Continuous variables were summarized as medians with interquartile ranges, and categorical variables as counts and percentages. Categorical variables were compared using the *χ* ^2^ test or Fisher's exact test, as appropriate. For comparisons among more than two groups, the Kruskal–Wallis test was used, followed by Dunn's post hoc test with a Holm correction for multiple comparisons when indicated (Figure 4). A two‐sided *p* value < 0.05 was considered statistically significant. All statistical analyses were performed using JMP Pro (SAS Institute, Cary, NC, USA).

## 3 Results

### 3.1 Effects of 3T MRI Scanning for Pacemakers

When the MRI mode was turned off, although conversion to the magnet mode and magnet episodes were recorded in some devices, no telemetry failure or reset were observed in the 3T‐conditional pacemakers after imaging. In addition, when the MRI mode was turned off and the device was set to the DDD mode, frequent activation of AMS and V pacing inhibition were observed, as expected (Table 4). Most of the 1.5‐T MRI‐conditional pacemakers also showed no adverse events. However, three of the 1.5‐T MRI‐conditional pacemakers with a low battery status exhibited telemetry communication failure after MRI scanning, despite having the MRI mode enabled (Table 5). Among them, two devices could no longer be reprogrammed and would have required urgent device replacement in a clinical setting. In the non–MRI‐conditional pacemaker group, five devices showed a reset in which the settings reverted to the default mode, and one device showed telemetry failure after MRI scanning (Table 6). Of these six devices, three also had a battery status approaching the elective replacement indicator (ERI). Although all devices that experienced reset could be reprogrammed appropriately, telemetry failure in one device was irreversible. When all pacemakers were compared according to battery status, pacemakers with a low battery status ≤ 30% (*n* = 11) experienced resets or telemetry failure more frequently than those with a higher battery status (*n* = 22) (> 30% vs. ≤ 30%: 3/22 [14%] vs. 6/11 [55%], *p* = 0.013) (Figure 2). In the non‐MRI‐conditional pacemaker group, interestingly even in the DDD mode, nearly all devices entered an asynchronous mode due to magnet mode activation, and none of the devices exhibited AMS. When the MRI mode was turned OFF in MRI‐conditional devices and in non–MRI‐conditional pacemakers, 16 devices programmed to an asynchronous mode (DOO or VOO) switched to the magnet mode, whereas 18 devices programmed to the demand mode (DDD/VVI) similarly switched to the magnet mode. There was no significant difference between the two modes (asynchronous vs. demand mode: 16/31 [52.0%] vs. 18/31 [58.0%], *p* = 0.610; Figure 3). Nine 3T MRI‐conditional pacemakers set to the DDD‐mode subsequently transitioned to AMS. These mode transitions varied by manufacturer, as described below.

**Table 4:** Adverse events across device settings following 3T MRI scanning of 3T‐conditional CIEDs.

3T TV‐pacemaker *N* = 19	DOO/VOOMRI mode on *N *= 19	DOO/VOOMRI mode off *N *= 19	DDD/VVIMRI mode off *N* = 19
Telemetry failure (%)	0 (0)	0 (0)	0 (0)
Reset (%)	0 (0)	0 (0)	0 (0)
Magnet episode (%)	0 (0)	5 (26.3)	6 (31.5)
Magnet rate pace (%)	0 (0)	10 (52.6)	11 (57.9)
Atrial mode switch (%)	N/A	0 (0)	9 (47.4)
Noise reversion (%)	N/A	0 (0)	1 (5.3)
Inhibition of V pacing (%)	N/A	0 (0)	4 (21.2)

3T TV‐ICD *N* = 8	DOO/VOOMRI mode on *N* = 8	DOO/VOOMRI mode off *N *= 8	DDD/VVIMRI mode off *N *= 8
Telemetry failure (%)	0 (0)	0 (0)	0 (0)
Reset (%)	0 (0)	0 (0)	0 (0)
Magnet episode (%)	0 (0)	1 (12.5)	1 (12.5)
Atrial mode switch (%)	N/A	0 (0)	6 (75.0)
V high rate (%)	N/A	2 (25.0)	2 (25.0)
Noise reversion (%)	N/A	0 (0)	0 (0)
Inhibition of V pacing (%)	N/A	0 (0)	6 (75.0)

**Table 5:** Adverse events across device settings following 3T MRI scanning of 1.5T‐conditional CIEDs.

1.5T TV‐pacemaker *N *= 6	DOO/VOOMRI modeon *N *= 6	DOO/VOOMRI mode off *N *= 4	DDD/VVIMRI mode off *N* = 4
Telemetry failure (%)	3 (50.0)	0 (0)	0 (0)
Reset (%)	0 (0)	0 (0)	0 (0)
Magnet episode (%)	0 (0)	3 (75.0)	3 (75.0)
Magnet rate pace (%)	0 (0)	0 (0)	0 (0)
Atrial mode switch (%)	N/A	0 (0)	0 (0)
Noise reversion (%)	N/A	0 (0)	1 (25.0)
Inhibition of V pacing (%)	N/A	0 (0)	2 (50.0)

1.5T TV‐ICD *N* = 4	DOO/VOOMRI mode on *N *= 4	DOO/VOOMRI mode off *N* = 4	DDD/VVIMRI mode off *N *= 4
Telemetry failure (%)	0 (0)	0 (0)	0 (0)
Reset (%)	0 (0)	0 (0)	0 (0)
Magnet episode (%)	0 (0)	2 (50.0)	1 (25.0)
Atrial mode switch (%)	N/A	0 (0)	3 (75.0)
V high rate (%)	N/A	0 (0)	1 (25.0)
Noise reversion (%)	N/A	1 (25.0)	1 (25.0)
Inhibition of V pacing (%)	N/A	0 (0)	3 (75.0)

**Table 6:** Adverse events across device settings following 3T MRI scanning of non‐MRI‐conditional CIEDs.

non‐MRI TV‐ICD *N *= 5	DOO/VOO *N *= 5	DDD/VVI *N *= 5
Telemetry failure (%)	0 (0)	0 (0)
Reset (%)	0 (0)	0 (0)
Magnet episode (%)	2 (40.0)	2 (40.0)
Atrial mode switch (%)	0 (0)	3 (60.0)
V high rate (%)	1 (20.0)	2 (40.0)
Noise reversion (%)	1 (20.0)	1 (20.0)
Inhibition of V pacing (%)	0 (0)	2 (40.0)

non‐MRI TV‐pacemaker *N *= 8	DOO/VOO *N *= 8	DDD/VVI *N *= 8
Telemetry failure (%)	0 (0)	1 (12.5)
Reset (%)	4 (50.0)	5 (62.5)
Magnet episode (%)	0 (0)	1 (12.5)
Magnet rate pace (%)	6 (75.0)	7 (87.5)
Atrial mode switch (%)	0 (0)	0 (0)
Noise reversion (%)	0 (0)	0 (0)
Inhibition of V pacing (%)	0 (0)	2 (25.0)

The four leadless pacemakers (Leadless‐PM) remained in their programmed mode without switching to the magnet mode during the MRI scan, and no malfunctions or alerts were observed. Furthermore, mild suppression of ventricular pacing was confirmed in all Leadless‐PMs during the VVI mode, with a V pacing ratio of 98.4% ± 0.82%.

### 3.2 Differences in Pacemaker Magnet‐Mode Responses Among Manufacturers

The influence of magnetic fields on CIED behavior during 3T MRI varied among manufacturers, as summarized below:

*Medtronic*: Transition to the magnet mode was observed in two out of six devices with the MRI mode turned off; however, this transition was not reproducible. Devices operating in the DDD mode switched to the AMS mode during scanning and returned to their originally programmed mode after imaging.

*Abbott (St. Jude Medical)*: Two out of 14 devices with the MRI mode turned off exhibited a transition to magnet mode at around 2 mT. Magnet‐response episodes were observed in all devices. In Abbott pacemakers, which have a magnet episode function, cases in which a magnet episode was recorded on the programmer but no magnet‐rate pacing was confirmed on the electrocardiogram were counted as magnet episodes only. Devices programmed in the DDD mode that did not switch to the magnet mode instead transitioned to the AMS mode during the MRI scan and subsequently returned to their originally programmed mode.

*Biotronik*: Devices switched to magnet mode at approximately 5 mT but returned to the programmed mode after about 10 beats. During the programmed mode, devices switched to AMS during scanning.

*Boston Scientific*: Devices switched to the magnet mode at magnetic field strengths of approximately 1–5 mT within the MRI suite. No AMS events were observed.

*Micro Port CRM (Sorin)*: Devices switched to the magnet mode at magnetic field strengths between approximately 10 and 40 mT and remained in the magnet mode throughout the scan.

### 3.3 Effects of 3T MRI Scanning for ICD

In this study, none of the ICDs tested (17 transvenous and 4 subcutaneous systems)—whether MRI‐conditional or non–MRI‐conditional—exhibited significant adverse events such as device reset or telemetry failure after the MRI examination. Telemetry remained available even at the ERI, allowing post‐scan reprogramming when needed.

Because magnet application in transvenous ICDs (TV‐ICD) typically suspends tachyarrhythmia therapy and does not provide asynchronous pacing as in pacemakers, we did not expect substantial pacing changes with magnetic field exposure. Nevertheless, magnet detection episodes were confirmed in five devices. Overall, exposure to the MRI environment resulted in little to no change in the pacing status in most devices. However, in twelve devices (nine 3T and 1.5T MRI–conditional and three non–MRI‐conditional), switching to AMS was observed during demand‐mode programming, and a ventricular tachycardia episode was recorded (two 3T MRI–conditional and three non–MRI‐conditional). Clear pacing inhibition was observed in eleven TV‐ICD devices (64.7%). According to the manufacturer's instructions for use, all tested S‐ICDs showed speaker impairment, with a decrease in sound level confirmed during the speaker test.

### 3.4 Suppression of Ventricular Pacing in Demand Mode During MRI

We compared pacing rate suppression among CIEDs that could be programmed to the demand mode by calculating the change in the ventricular pacing ratio (ΔVp) from asynchronous pacing with MRI mode OFF. A comparison of pacing inhibition among transvenous pacemakers (TV‐PM), TV‐ICD, and leadless pacemakers (leadless PM) demonstrated significant differences among the three groups and specifically between TV‐PMs and TV‐ICDs (TV‐PM vs. TV‐ICD vs. leadless PM: 0% [0–0] vs. 7% [1.0–33.0] vs. 1.45% [0.83–2.13]; *p* < 0.001; Figure 4).

**Figure 5:**
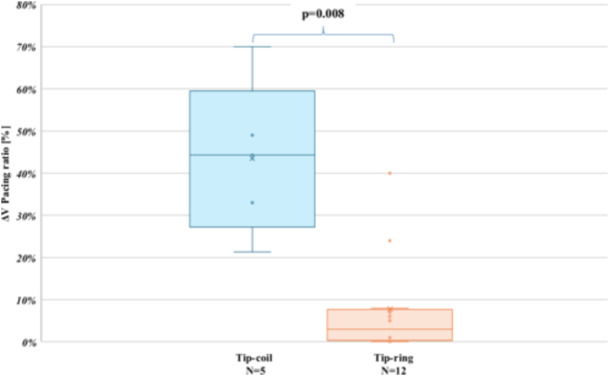
Box‐and‐whisker plot comparing pacing suppression according to sensing polarity in transvenous ICDs. Pacing suppression was significantly greater with tip‐coil sensing than with tip‐ring sensing (44.3% [27.3–59.5] vs. 3.0% [0.5–7.5], respectively; *p* = 0.008). ΔVp was defined as the difference in pacing percentage between asynchronous and demand modes.

Furthermore, when investigating the degree of pacing suppression in TV‐ICDs according to the sensing polarity (tip‐coil vs. tip‐ring), tip‐coil showed greater suppression than tip‐ring, with a significant between‐group difference (44.3% [27.3–59.5] vs. 3.0% [0.5–7.5], respectively; *p* = 0.008; Figure 5).

## 4 Discussion

### 4.1 Major Findings

The following important findings were obtained in this in vitro study. First, some non‐3T MRI–compatible pacemakers with battery levels approaching the ERI exhibited telemetry failure or complete parameter reset after 3T MRI scanning. In particular, two 1.5T MRI‐conditional devices with the MRI mode turned on and one non–MRI‐conditional device developed irreversible telemetry failure after the MRI scan. These telemetry failures and resets occurred at a significantly higher frequency in devices with low battery levels. Second, when the MRI mode was turned off, some CIEDs switched to the Magnet mode or AMS depending on the manufacturer and pacing configuration, and the frequency of magnet mode activation occurred regardless of whether the device was in the asynchronous mode or demand mode. Third, in 3T‐conditional pacemakers and ICDs, no resets or telemetry failures occurred. On the other hand, the frequency of ventricular pacing suppression in demand mode was higher in ICDs. Finally, 3T MRI did not result in any severe electrical malfunctions that would necessitate device replacement in the clinical setting in most non–3T‐compatible CIEDs, particularly when programmed to asynchronous pacing modes. These observations suggest that, with careful pre‐scan evaluation and appropriate device programming, 3T MRI may be feasible in selected non–3T‐compatible systems under controlled conditions.

### 4.2 Resetting and Telemetry Failure After 3T MRI Scanning

Historically, device resets during MRI examinations were primarily observed in early‐generation systems, particularly Medtronic devices implanted before 2005 [2, 7, 12]. Although advances in device technology have progressively improved the electromagnetic tolerance of CIEDs, such malfunctions have not been completely eliminated. A relatively recent multicenter study [3] reported a case of inaccurate battery status fault codes that required generator replacement, even in relatively modern device, suggesting that electrical instability can still occur under certain MRI conditions. Notably, episodes of complete device reset leading to cardiac arrest have been documented during 3T MRI scanning, even with “modern” CIED systems manufactured after 2000 [9].

In the present study, 1.5T MRI‐conditional CIEDs approaching the ERI stage exhibited telemetry failure and unintended resetting of programmed parameters after 3T MRI scanning, even when the MRI mode was activated. When the MRI mode is ON, these devices operate with pacing outputs higher than those used in routine clinical settings, resulting in greater energy consumption. Therefore, in generators already near battery depletion, the combination of a reduced residual capacity and exposure to strong magnetic fields may have contributed to telemetry loss and/or parameter resetting.

Most manufacturers restrict or prohibit the activation of the MRI mode when the device battery reaches the ERI stage. Biotronik specifies that the MRI mode is not recommended when battery capacity falls below 30% [13]. Although several guidelines [6, 14] state that a depleted battery status is considered high risk, there are few studies that specifically focus on this topic. Okamura et al. [15] showed that patients with pacemakers and ICDs with a nearly depleted battery can safely undergo MRI when patients are not pacemaker dependent. However, they also showed that two scans with pacemakers close to the ERI resulted in a power‐on‐reset in patients. Their study demonstrated the risk associated with older non–MRI‐conditional devices; however, our study is important in that it shows similar outcomes may occur even in the MRI mode when the battery level is low.

### 4.3 Switching to Magnet Mode and Potential Patient Impact

Current ESC guidelines recommend that, for MRI non‐compatible devices, asynchronous pacing modes such as DOO or VOO should be selected in pacing‐dependent patients, while those with intrinsic heart rates exceeding 50 bpm may be programmed to DDI, VVI, or AAI modes during MRI scanning [6]. On the other hand, the present experiment suggests that the potential for magnet mode activation should always be considered, even when the device is programmed to an asynchronous mode. If the intrinsic rate competes with the pacing rate or magnet rate, such interference may lead to palpitations, hemodynamic instability, or even induction of serious arrhythmias. Therefore, it is essential to evaluate the patient's intrinsic rhythm and programmed pacing rates in advance to minimize the risk of adverse events during MRI procedures.

In addition, this study demonstrated that both the susceptibility to entering the magnet mode and its activation vary substantially among manufacturers. These manufacturer‐specific differences in MRI mode behavior are likely related, at least in part, to differences in the threshold for magnetic field intensity. Awareness of these differences in advance is clinically very important, as it allows appropriate measures—such as adjustment of the programmed pacing rate—to be taken.

### 4.4 Switching to AMS and Pacing Inhibition

Some CIEDs that were not triggered to the magnet mode by the magnetic field during DDD pacing, instead transitioned to AMS or pacing inhibition during 3T MRI scanning. This phenomenon was likely due to oversensing of magnetic field–induced noise on the atrial and ventricular leads, which was misinterpreted by the device as atrial fibrillation or own beats. Such inappropriate oversensing may suppress pacing output—potentially leading to asystole. These findings suggest that oversensing during 3T MRI may pose a risk of serious adverse events, depending on the device's function and programmed mode. Currently, for non–MRI‐conditional devices, the DDD mode is generally avoided during MRI examinations, as noted above. However, it is important to recognize that similar malfunctions could occur if a device were inadvertently brought into the MRI environment without being switched to the MRI mode or if device reprogramming were unintentionally omitted.

The susceptibility to AMS and pacing inhibition depends on the magnet mode, and because the magnet mode behavior differs among manufacturers, as described above, it is important to understand that manufacturer‐specific characteristics are consequently associated with the occurrence of AMS and pacing inhibition.

Although only four leadless pacemakers were evaluated, precluding definitive conclusions, the Micra does not have a magnet mode; pacing inhibition in demand mode was mild, and the small size of both the device and the electrode may limit the effects of the MRI magnetic field.

### 4.5 Characteristics of ICD Responses Within a 3T MRI Environment

Although there has been a report [1] of generator failure requiring immediate replacement in ICDs, the results of the present study suggest that, because ICDs inherently lack a pacemaker‐like magnet mode, the likelihood of electrical malfunction is low when the device is programmed to an asynchronous mode. On the other hand, as shown in Figure 4, suppression of ventricular pacing in demand mode was greater in ICDs. This phenomenon is presumed to occur because, as noted above, ICDs do not have a magnet mode; therefore, pacing does not become asynchronous in demand mode, leading to oversensing of MRI‐related noise and consequent pacing inhibition.

The impact of the battery status in ICDs could not be determined definitively due to the small number of devices evaluated; however, consistent with a previous clinical study [15], none of the 17 TV‐ICDs examined in this study experienced resets or telemetry failure.

Furthermore, among sensing configurations that used vectors incorporating a defibrillation coil, pacing inhibition was greater with coil‐inclusive sensing. The larger effective sensing electrode surface area in coil‐inclusive configurations may increase susceptibility to magnetic field–related interference, as suggested by our findings.

### 4.6 Clinical Implications

**Figure 6:**
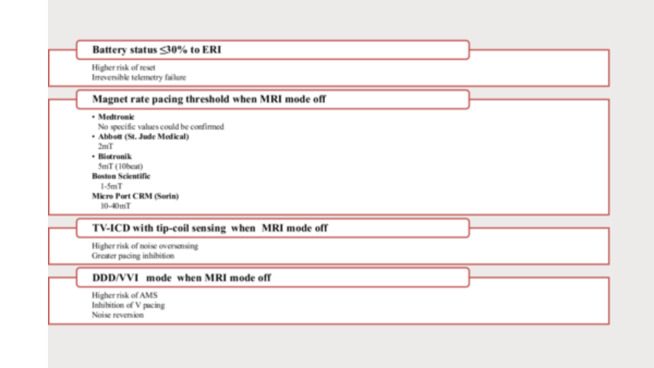
The clinically important points that require particular caution in this study are summarized in Figure. AMS, atrial mode switch; ERI, elective replacement indicator; mT, milli Tesla; TV‐ICD, transvenous implantable cardiac defibrillator.

Our findings indicate that particular caution is required when performing 3T MRI in patients with non–3T MRI‐conditional devices, with the following considerations: (1) devices with low battery status require careful attention because of the risk of reset or irreversible telemetry failure; (2) with MRI mode OFF, even devices programmed to asynchronous mode may switch to magnet mode, potentially resulting in competition with the intrinsic rhythm; (3) TV‐ICDs with tip‐to‐coil sensing and MRI mode OFF may carry a high risk of pacing inhibition; and (4) in demand‐mode pacemakers with MRI mode OFF, pacing inhibition or programming changes such as AMS may occur (Figure 6).

Current international guidelines and statements [5, 6, 16] have increasingly expanded the indications for MRI in patients with CIEDs, often making the practical precautions less clearly defined. In this context, the present study is of substantial significance in identifying specific issues that warrant attention. At the same time, our findings also suggest an important clinical implication: provided that sufficient attention is paid to these considerations, and that device settings such as demand mode—which would generally be avoided during MRI—are not used, 3T MRI appears feasible, with few cases resulting in severe malfunction necessitating device replacement.

At present, MRI in patients with implanted devices involves increasingly complex combinations of device types and programming configurations, and in clinical practice, some cases require individualized adjustments and careful procedural planning [17]. The present findings are expected to serve as a useful reference to support clinical decision‐making for MRI in this increasingly complex setting.

### 4.7 Study Limitations

This study was a small‐scale, single‐center in vitro experiment. Therefore, we were unable to evaluate potential effects that might be observed in clinical studies, such as heating effects and changes in pacing thresholds. In particular, 3T MRI has the potential for higher SAR compared with 1.5T MRI, which is an important consideration; however, this was not evaluated in the present study.

In addition, the range of CIEDs manufacturers, device models, and MRI scanning conditions was limited, and the evaluation period was short. The sample size for certain manufacturers (e.g., MicroPort/Sorin or Leadless PMs) was too small to definitively conclude that their magnet‐mode behavior is representative of all their device models. In particular, although we demonstrated the risk associated with a low battery status in this study, the limited number of devices prevented us from determining a cutoff value. Consequently, the clinical outcomes for patients and the long‐term effects on CIED parameters remain undetermined.

## 5 Conclusion

In direct in vitro 3T MRI exposure experiments involving non–3T MRI CIEDs, some pacemakers approaching ERI demonstrated an increased risk of telemetry failure or reset, and those with MRI mode turned off showed a non‐negligible risk of mode switching. However, the majority of devices did not exhibit severe electrical malfunctions that would necessitate replacement in a clinical setting, and no instances of device reset or telemetry failure were observed in ICDs.

## Funding

The authors have nothing to report.

## Ethics Statement

Ethical approval was not required as this study involved only in vitro devices without human data.

## Consent

The requirement for informed consent was waived because this was an in vitro device study without human participants or identifiable data.

## Conflicts of Interest

Y.I. received lecture fees from Medtronic, Japan and Biotronik, Japan. The Department of Cardiology received grant support from Boston Scientific, Japan and Abbott Medical, Japan. Other authors declare no conflicts of interest.

## Data Availability

The data underlying this article will be shared upon reasonable request to the corresponding authors.
